# On the Use of Nitrate of Silver

**Published:** 1900-06

**Authors:** H. R. Neeper


### Domestic Correspondence.

ON THE USE OF NITRATE OF SILVER.

To the Editor:

Sir,—Apropos of Dr. J. Morgan Howe's article, "Notes on Nitrate of Silver," the method of application is of importance. In numerous cases, some of which are mentioned in the paper, the conditions presenting where the nitrate of silver treatment is indicated make the application of a caustic a serious matter, because of certain difficulties. The greatest of these is keeping these surfaces dry, as the salt is so quickly dissolved by contact with saliva that the gum is usually cauterized also. I read somewhere that by heating the end of a German silver probe to nearly or quite red heat, and dipping in the crystals of the salt, some little would fuse on the probe, making it an ideal carrier and applicator. I have used it thus many times during the last year with great satisfaction. If Dr. Howe would give us the details of how he uses the caustic, it would be thankfully received.

H. R. Neeper.

